# Areal bone mineral density, trabecular bone score and 3D-DXA analysis of proximal femur in psoriatic disease

**DOI:** 10.3389/fmed.2024.1341077

**Published:** 2024-01-30

**Authors:** Eric Toussirot, Renaud Winzenrieth, François Aubin, Daniel Wendling, Charline Vauchy, Maxime Desmarets

**Affiliations:** ^1^INSERM CIC-1431, Centre d’Investigation Clinique, Pôle Recherche, CHU de Besançon, Besançon, France; ^2^Rhumatologie, Pôle PACTE (Pathologies Aiguës Chroniques Transplantation Éducation), CHU de Besançon, Besançon, France; ^3^Département Universitaire de Thérapeutique, Université de Franche-Comté, Besançon, France; ^4^UMR 1098 RIGHT, INSERM, Établissement Français du Sang, Université de Franche-Comté, Besançon, France; ^5^3D-SHAPER Medical, Barcelona, Spain; ^6^Dermatologie, Pôle PACTE (Pathologies Aiguës Chroniques Transplantation Éducation), CHU de Besançon, Besançon, France; ^7^EA 4266 EPILAB, Université de Franche-Comté, Besançon, France

**Keywords:** DXA, trabecular bone score, bone microarchitecture, 3D DXA analysis, psoriatic arthritis, psoriasis, osteoporosis

## Abstract

**Objectives:**

To evaluate bone mineral density (BMD) and bone quality, with assessment of the cortical and trabecular compartments, in patients with psoriasis (PsO) alone or with psoriatic arthritis (PsA).

**Methods:**

Patients with PsA and patients with PsO alone were evaluated and compared to control subjects matched for age, sex and body mass index category. Areal BMD (aBMD) was determined for the lumbar spine, femoral neck, total hip and total body using dual-energy X-ray absorptiometry (DXA). Bone quality was evaluated by using trabecular bone score (TBS) at the lumbar spine, and by 3D DXA-based analysis (3D Shaper) for the proximal femur.

**Results:**

One hundred ninety-six subjects including 52 patients with PsA and 52 patients with PsO and their respective paired controls were analyzed. Patients with PsA had comparable aBMD, TBS and 3D DXA analysis parameters compared to their paired controls. After adjustment for confounders, patients with PsO alone were characterized by a higher aBMD at the left femur and higher cortical 3D DXA derived parameters (total hip cortical surface BMD and total hip cortical thickness) than their paired controls. TBS was decreased in PsO compared to their controls.

**Conclusion:**

Patients with PsA had normal bone mass and bone quality parameters. Patients with PsO were characterized by higher femoral neck bone density by DXA and cortical parameters by 3D DXA-based analysis, supporting no increased risk for hip fracture. Conversely, bone texture by TBS assessment was decreased in patients with PsO, which may be associated with impaired vertebral bone resistance.

## Introduction

Psoriasis (PsO) is a chronic immune-mediated disease of the skin characterized by abnormal expression of keratinocytes and infiltration of the dermis with dendritic cells, macrophages, neutrophils and T lymphocytes (1). Around 30% of the patients with PsO also develop psoriatic arthritis (PsA) with axial and/or peripheral manifestations (2). Both PsO and PsA share similar Th1-and Th17-driven inflammation with increased production of inflammatory cytokines, including TNF-alpha, IFN-gamma, IL-6, IL-8, IL-17 and IL-23 in the skin and the synovial membrane (3). Osteoporosis is a systemic bone disease characterized by diminished bone mass and altered bone microarchitecture, leading to decreased bone strength and an increased risk of fragility fracture. The question of bone involvement in psoriatic disease has led to specific studies evaluating bone mass and the risk of fractures. However, the evidence for bone impairment in psoriatic disease remains debated. Indeed, a systematic review found that studies were heterogeneous, with inconsistent results (4). In addition, a recent meta-analysis concluded that there is no increased risk of osteoporosis in patients with psoriatic disease (5). Conversely, an increased risk of fracture was observed in a cross-sectional study from an emergency department in the United States (6) and in a population-based cohort study in the United Kingdom (7). These discrepancies may result from heterogeneous study populations, sample sizes, various study designs, and limiting of bone assessment to bone mineral density (BMD) measurement using the reference method, namely dual-energy X-ray absorptiometry (DXA). One study evaluated quantitative and qualitative changes in the bone in psoriatic patients using high-resolution peripheral quantitative computed tomography (HR-pQCT) and found decreased trabecular BMD and bone microstructure in PsA (8). In addition, the trabecular bone score (TBS), an indirect method of evaluating bone microarchitecture, has rarely been used in psoriatic disease (9). Finally, besides BMD, other parameters may determine bone quality, such as bone geometry, microarchitecture and 3D distribution of bone material. 3D-Shaper modelling, a recently developed method for non-invasive bone structure assessment using DXA images, provides a detailed analysis of the proximal femur, including separate measurements of the cortical layer and trabecular compartments. It provides additional information on the bone macrostructure and bone strength, and could enhance understanding of bone fragility and fracture risk (10–13).

In this study, we aim to evaluate the impact of systemic inflammation on bone health by evaluating bone mass and bone quality, with assessment of the cortical and trabecular compartments, using DXA, TBS and 3D-analysis of the proximal femur in patients with PsO alone or with PsA.

## Patients and methods

### Patients

The selection of cases and controls, and the validation of diagnosis have previously been described in the ADIPSO study (14). Briefly, patients with PsO alone or patients with PsA were consecutively enrolled at the University Hospital of Besançon, France. Control subjects were recruited among the hospital staff (healthy subjects) and among patients referred to the rheumatology department for mechanical back pain. Each patient was matched to a single control subject according to age, sex and body mass index (BMI). Exclusion criteria were age younger than 18 years or older than 80 years, patients receiving a biological agent or systemic corticosteroids at a dose exceeding 10 mg prednisone per day. For all subjects, socio-demographic data, BMI (weight (in kg)/square of the height (in metres)), disease duration, menopausal status, smoking habits, current treatment for PsO and PsA (topical treatments, systemic corticosteroids, systemic conventional agents) were available. The psoriasis area severity index (PASI) was used to evaluate the extent and severity of skin involvement and the dermatology life quality index (DLQI) was recorded. In the PsA group, disease activity was evaluated by disease activity score 28 joint-erythrocyte sedimentation rate (DAS28-ESR), Bath ankylosing spondylitis disease activity index (BASDAI) and composite psoriatic disease activity index (CPDAI), including 66/68 joint count. The health assessment questionnaire (HAQ) and ankylosing spondylitis quality of life (ASQoL) were also obtained. Enthesitis and dactylitis were evaluated using the Leeds Enthesitis Index and dactylitis count, respectively. The systematic coronary risk evaluation SCORE was used to evaluate cardiovascular risk of the patients. Previous history of low-trauma fracture was recorded. Written informed consent was obtained from each participant and the study was approved by the local Ethics Committee (*Comité de Protection des Personnes CPP Est-II*, reference number: 13/411).

### Bone mineral density

Areal BMD (aBMD) was evaluated by DXA using a Lunar densitometer (iDXA; GE Healthcare, Madison, WI, United States). Measurements were performed following standard technical procedures and according to the manufacturer’s instructions. The DXA device was stable during the study period and all measurements were performed by experienced technicians. A single DXA device was used during the study. The following regions were evaluated: (i) lumbar spine (LS; L2 to L4, anteroposterior view); (ii) hip: femoral neck (FN) and total hip (TH) with measurements from both sides; results were given as mean of both sides and for left and right sides; (iii) total body skeleton. Results were expressed as g/cm^2^ and T score. For specific measurements at each hip, results were only expressed as g/cm^2^. Quality control scans were performed daily during the study period using the manufacturer’s standards. The coefficients of variation for the LS, FN, TH and total body scan were 1.0, 1.5, 0.9 and 0.7%, respectively.

### Trabecular bone score

The TBS was calculated from anteroposterior L2-L4 BMD images using TBS iNsight V1.8 (Med-Imaps, Pessac, France). In each region of measurement (L2 to L4), the TBS was evaluated based on a grey-level analysis of DXA scan, as previously described (15). The coefficient of variation for TBS was 1.5%.

### Proximal femur 3D reconstruction

3D-DXA analysis was performed using 3D Shaper software (version 2.10, 3D-SHAPER Medical, Barcelona Spain). Briefly, the software uses an algorithm based on a 3D statistical shape and density model of the proximal femur built from a QCT scan database of Caucasian men and women (16). The 3D model is registered onto the DXA scan to obtain a 3D QCT-like patient-specific model of the proximal femur. The cortex is subsequently segmented and the trabecular bone part is extracted. More details about the algorithm can be found elsewhere (10). The parameters considered for the current study were: integral (cortical + trabecular) volumetric BMD (integral vBMD; mg/cm^3^), cortical surface BMD (cortical sBMD; mg/cm^2^), cortical thickness (Cth; mm), trabecular volumetric BMD (trabecular vBMD; mg/cm^3^), and cortical volumetric BMD (cortical vBMD; mg/cm^3^). In addition, for cortical sBMD and Cth, we considered measurements at specific regions of interest: neck, intertrochanteric and shaft regions. Analysis was performed on the FN and TH and results were given for each proximal femur. Coefficients of variation for cortical sBMD, Cth, trabecular vBMD, cortical vBMD were 1.5, 1.5, 4.5 and 1.7%, respectively. Hip DXA scans were sent to 3D-SHAPER Medical where analysis was performed by a trained specialist (RW) who was blinded to the patient group.

### Statistical analysis

Patients and controls were described using mean ± standard deviation (SD) for quantitative variables and frequencies and percentage for categorical variables. Comparisons between paired cases and controls (patients with PsA and their paired controls on the one hand, and patients with PsO and their paired controls on the other hand) were performed using paired Student *t*-tests for quantitative variables and McNemar’s test for categorical variables. Comparison of bone parameters (DXA measurements, TBS and proximal femur 3D-analysis) was also performed using a multivariate linear model (ANCOVA) with adjustment for for main factors influencing bone mass, i.e., age, BMI and a composite of gender and menopausal status (male/female non-menopausal/female menopausal). The correlation between TBS and measurements of disease extent and/or activity of the patients with PsO (PASI, ESR, CRP and IL-6) was analyzed using Pearson’s coefficient. The correlation between DXA measurements at the femur and 3D-analysis parameters of the proximal femur were analyzed with the same method. Statistical analyses were performed by the research methods unit (uMETh) of the clinical investigation center, University Hospital of Besancon, using SAS version 9.4 (SAS Institute Inc., Cary, NC, United States). The significance level was set at *p* < 0.05. Considering the exploratory nature of the study, no adjustments for multiple comparisons were made.

### Post-hoc power analysis

The ADIPSO study was powered to detect differences in visceral adiposity in PsA patients (14). Power analysis indicates that 52 pairs of patients provide 94% power to detect a medium (*d* = 0.5) effect size but only 29% power to detect a small effect size (*d* = 0.2) according to Cohen (17).

## Results

One hundred and 96 Caucasian subjects were analyzed, namely: 52 patients with PsA, 52 patients with PsO alone and 92 controls (a limited number of subjects (*N* = 12) were used as controls for both PsA and PsO patients for matching reasons). Demographics of the study population are presented in Table 1. Patients with PsA had an oligo or polyarticular form. There were no differences between patients with PsA and patients with PsO and their respective controls concerning age, gender, BMI, smoking and menopausal status. Patients with PsO had a longer disease duration compared to patients with PsA (*p* < 0.0001). The proportion of menopausal women in the PsO group was numerically lower than in the control group, but without reaching statistical significance (*p* = 0.32). Eighty percent of patients with PsA and 36.5% with PsO were receiving conventional/systemic agents at the time of assessment and they were all biologic-naïve. The proportion of patients receiving systemic corticosteroids was low in the PsA group (15.4%; mean daily dose: 5.8 ± 2.5 mg), and zero in the PsO group. Disease activity (based on DAS28-ESR, BASDAI and CPDAI) was moderate or high in the PsA group and only 25% of patients had minimal disease activity. Laboratory parameters of inflammation (ESR, CRP and IL-6) were significantly elevated in PsA patients versus their controls, while only ESR differed significantly between PsO patients and their controls (Table 1). No patient from the PsO and PsA groups or among the control subjects had an anti-osteoporotic medication (bisphosphonates, denosumab or teriparatide) at the time of assessment or had previously had a fragility fracture.

**Table 1:** Clinical characteristics and laboratory parameters of disease activity in patients with psoriatic arthritis or psoriasis alone, and their paired controls.

	PsA mean ± SD or *N* (%)	PsA controls mean ± SD or *N* (%)	*p*	PsO mean ± SD or N (%)	PsO controls mean ± SD or N (%)	*p*
N	52	52		52	52	
Age (years)	52.5 ± 11.7	52.8 ± 11.1	0.69	50.5 ± 12.8	50.7 ± 12.8	0.76
Gender M/F	25/27 (48/52)	25/27 (48/52)	-	38/14 (73/27)	36/16 (69/31)	0.16
Disease duration (years)	9.1 ± 6.7	–	-	18.1 ± 13.8	-	-
BMI (kg/m^2^)	27.4 ± 5.9	27.7 ± 6.4	0.74	28.4 ± 5.8	28.2 ± 6.1	0.72
Menopausal women	18/27 (66.7)	16/27 (59.3)	0.32	5/14 (35.7)	8/16 (50.0)	0.32
Smoking Never Former Current	32 (61.5) 11 (21.2) 8 (15.4)	35 (67.3) 6 (11.5) 10 (19.2)	0.69	25 (48.1) 9 (17.3) 18 (34.6)	28 (53.8) 14 (26.9) 9 (17.3)	0.09
SCORE Treatments Systemic GC Topical MTX LFM SSZ Retinoids Cyclosporin	1.39 (1.89) 8 (15.4) 3 (5.7) 37 (71.2) 4 (7.7) 1 (1.9) 0 0	–		1.53 (1.53) 0 4 (7.7) 15 (28.8) 0 0 3 (5.8) 1 (1.9)	–	
DAS-28-ESR	3.49 ± 1.48	–		–	–	
BASDAI	4.67 ± 3.8	–		–	–	
CPDAI	7.42 ± 3.34	–		–	–	
PASI	2.43 ± 4.1	–		8.4 ± 4.9	–	
Leeds enthesitis index	1.3 ± 1.5	–		–	–	
MDA	13 (25)	–		–	–	
HAQ	0.75 ± 0.7	–		–	–	
ASQOL	8.2 ± 6.4	–		–	–	
DLQI	3.9 ± 5.4	–		10.4 ± 7.1	–	
ESR (mm/h)	19.8 ± 16.3	6.9 ± 5.8	**< 0.0001**	10.8 ± 8.8	6.4 ± 6.5	**0.003**
CRP (mg/L)	10.5 ± 11.7	4.0 ± 4.8	**0.0003**	6.0 ± 9.0	4.7 ± 5.5	0.38
IL-6 (pg/ml)	10 ± 15.1	3 ± 2.2	**0.0014**	3.6 ± 3.1	2.9 ± 2.4	0.22

PsA, psoriatic arthritis; PsO, psoriasis; M, male; F, female; BMI, body mass index; GC, glucocorticoids; MTX, methotrexate; LFM, leflunomide; SSZ, sulfasalazine; MDA, minimal disease activity; DAS28-ESR, DAS 28 joint-erythrocyte sedimentation rate; CPDAI, composite psoriatic disease activity index; PASI, psoriasis area severity index; ASQoL, Ankylosing spondylitis quality of life; DLQI, dermatology life quality index; ESR, erythrocyte sedimentation rate; CRP, C-reactive protein; IL-6, interleukin-6. Bold values correspond to significant results.

Bone measurement results are presented in Table 2. aBMD and T score at the LS, FN, TH and total body was comparable between patients from the PsA group and their paired controls (with no difference between the right or left femur and the ipsilateral femur from the controls). In the PsO group, aBMD at the FN was higher in patients than in their controls, a result that was only significant on the left side (*p* = 0.03). However, when evaluating the mean of both hips, results for aBMD and T score were comparable between PsO patients and their controls. Total body aBMD was also higher in PsO patients (*p* = 0.05) while the corresponding T score did not differ between the patients and their paired controls. For the other regions analyzed (LS, TH), aBMD as well as T score were similar between PsO patients and their controls (Table 2).

**Table 2:** Areal bone mineral density, trabecular bone score and proximal femur DXA-based 3D analysis in patients with PsA or PsO and their paired controls.

		PsA mean ± SD	PsA controls mean ± SD	*p*	PsO mean ± SD	PsO controls mean ± SD	*p*
DXA parameters							
LS (L2-L4) aBMD (g/cm^2^)		1.27 ± 0.49	1.2 ± 0.2	0.32	1.25 ± 0.21	1.2 ± 0.18	0.15
LS (L2-L4) T Score		0.14 ± 1.75	0.07 ± 1.66	0.80	0.37 ± 1.69	0.07 ± 1.43	0.21
FN (mean) aBMD (g/cm^2^)		0.98 ± 0.23	0.95 ± 0.14	0.43	0.99 ± 0.15	0.97 ± 0.15	0.28
FN (mean) T score		−0.19 ± 1.81	−0.4 ± 1.1	0.42	−0.16 ± 1.1	−0.3 ± 1.2	0.35
FN aBMD (g/cm^2^) - Left		0.96 ± 0.16	0.93 ± 0.12	0.48	1.0 ± 0.14	0.97 ± 0.13	**0.03**
- Right		0.97 ± 0.16	0.94 ± 0.14	0.34	0.98 ± 0.15	0.98 ± 0.15	0.27
TH (mean) aBMD (g/cm^2^)		1.01 ± 0.16	1 ± 0.15	0.69	1.11 ± 0.22	1.06 ± 0.21	0.31
TH T score		−0.04 ± 1.26	−0.1 ± 1.21	0.73	0.33 ± 1.19	0.10 ± 1.28	0.18
TH aBMD (g/cm^2^) - Left		1.02 ± 0.17	0.99 ± 0.15	0.43	1.00 ± 0.14	0.97 ± 0.13	0.09
- Right		1.02 ± 0.17	1.00 ± 0.14	0.47	0.98 ± 0.15	0.98 ± 0.15	0.25
Total body aBMD (g/cm^2^)		1.2 ± 0.15	1.17 ± 0.14	0.22	1.25 ± 0.14	1.22 ± 0.14	**0.05**
Total body T score		0.83 ± 1.37	0.81 ± 0.98	0.63	0.91 ± 1.08	0.89 ± 1.05	0.76
TBS							
L2-L4 TBS		1.32 ± 0.12	1.32 ± 0.14	0.95	1.24 ± 0.15	1.3 ± 0.16	**0.006**
DXA-based 3D parameters							
Left femur	Integral TH vBMD (mg/cm^3^)	333.4 ± 71.1	314. 7 ± 60.3	0.49	342.4 ± 59.2	329.2 ± 59.2	0.12
	Integral neck vBMD (mg/cm^3^)	350.8 ± 73.2	334.6 ± 59.8	0.41	349.7 ± 60.8	341.2 ± 59.7	0.15
	TH cortical sBMD (mg/cm^2^)	169.7 ± 32.8	163.05 ± 29.7	0.73	182.0 ± 30.7	173.4 ± 30.3	**0.04**
	Neck cortical sBMD (mg/cm^2^)	135.3 ± 26.9	129.7 ± 21.9	0.61	140.3 ± 24.2	134.6 ± 22.5	**0.045**
	Intertroch cortical sBMD (mg/cm^2^)	162.3 ± 31.1	155.3 ± 29.3	0.69	171.4 ± 29.1	163.1 ± 28.9	**0.03**
	Shaft cortical sBMD (mg/cm^2^)	269.2 ± 49.0	257.2 ± 47.3	0.50	286.2 ± 47.5	272.7 ± 45.2	**0.04**
	TH Cth (mm)	1.99 ± 0.2	2.02 ± 0.2	0.22	2.11 ± 0.2	2.07 ± 0.2	0.06
	Neck Cth (mm)	1.63 ± 0.2	1.66 ± 0.2	0.27	1.72 ± 0.2	1.67 ± 0.2	0.08
	Intertroch Cth (mm)	1.91 ± 0.2	1.94 ± 0.2	0.16	2.03 ± 0.2	1.97 ± 0.2	**0.04**
	Shaft Cth (mm)	2.98 ± 0.3	3.01 ± 0.3	0.39	3.19 ± 0.4	3.09 ± 0.3	**0.03**
	TH trabecular vBMD (mg/cm^3^)	179.9 ± 48.9	169.4 ± 43.4	0.49	187.7 ± 43.1	180.1 ± 43.1	0.15
	Neck trabecular vBMD (mg/cm^3^)	2,211 ± 55.2	211.1 ± 49.2	0.40	222.7 ± 46.0	217.2 ± 48.4	0.18
	TH cortical vBMD (mg/cm^3^)	849.2 ± 112.1	803.8 ± 95.9	0.19	857.4 ± 87.2	837.3 ± 96.9	0.20
	Neck cortical vBMD (mg/cm^3^)	836.4 ± 103.0	792.6 ± 79.2	0.11	828.8 ± 78.9	815.1 ± 79.8	0.20
Right femur	Integral TH vBMD (mg/cm^3^)	322.8 ± 66.3	320.0 ± 58.7	0.80	333.4 ± 58.6	322.8 ± 56.0	0.17
	Integral neck vBMD (mg/cm^3^)	345.9 ± 71.7	338.5 ± 66.5	0.55	340.8 ± 61.5	335.3 ± 64.7	0.28
	TH cortical sBMD (mg/cm^2^)	168.4 ± 29.2	166.2 ± 29.2	0.85	177.3 ± 30.0	171.3 ± 29.6	0.11
	Neck cortical sBMD (mg/cm^2^)	135.0 ± 24.1	132.1 ± 24.9	0.77	136.6 ± 23.9	132.5 ± 25.5	0.12
	Intertroch cortical sBMD (mg/cm^2^)	160.8 ± 26.8	158.2 ± 29.7	0.87	166.9 ± 28.3	160.65 ± 29.6	0.07
	Shaft cortical sBMD (mg/cm^2^)	267.2 ± 44.4	263.0 ± 44.7	0.60	279.6 ± 47.3	271.3 ± 44.4	0.15
	TH Cth (mm)	2.01 ± 0.2	1.99 ± 0.1	0.72	2.08 ± 0.2	2.04 ± 0.2	0.06
	Neck Cth (mm)	1.66 ± 0.2	1.65 ± 0.2	0.64	1.68 ± 0.2	1.65 ± 0.2	0.10
	Intertroch Cth (mm)	1.93 ± 0.2	1.92 ± 0.2	0.52	1.99 ± 0.2	1.94 ± 0.2	**0.01**
	Shaft Cth (mm)	3.01 ± 0.3	2.99 ± 0.3	0.96	3.13 ± 0.4	3.07 ± 0.3	0.11
	TH trabecular vBMD (mg/cm^3^)	173.3 ± 46.9	170.6 ± 41.0	0.70	182.6 ± 43.7	176.0 ± 39.7	0.16
	Neck trabecular vBMD (mg/cm^3^)	218.8 ± 57.4	211.7 ± 51.3	0.42	215.7 ± 41.8	214.4 ± 49.2	0.37
	TH cortical vBMD (mg/cm^3^)	835.3 ± 102.5	828.6 ± 98.9	0.57	847.57 ± 86.8	837.2 ± 98.7	0.54
	Neck cortical vBMD (mg/cm^3^)	826.0 ± 92.6	815.8 ± 90.0	0.46	822.8 ± 77.3	814.9 ± 90.6	0.48

DXA, dual-energy X-ray absorptiometry; LS aBMD, lumbar spine (L2-L4) areal bone mineral density; FN aBMD, femoral neck areal bone mineral density; TH aBMD, total hip areal bone mineral density; TBS, trabecular bone score; TH, total hip; sBMD, surface bone mineral density; vBMD, volumetric bone mineral density; Cth, cortical thickness; intertroch, intertrochanteric region. Bold values correspond to significant results.

Trabecular bone score did not differ between patients with PsA and their controls, whereas TBS was significantly lower in PsO patients than in their paired controls (*p* = 0.006). TBS of patients with PsA under systemic corticosteroids (*N* = 8) were compared to their paired controls and the results showed no significant difference (1.24 ± 0.11 vs. 1.29 ± 0.1; *p* = 0.35).

**Figure 1:**
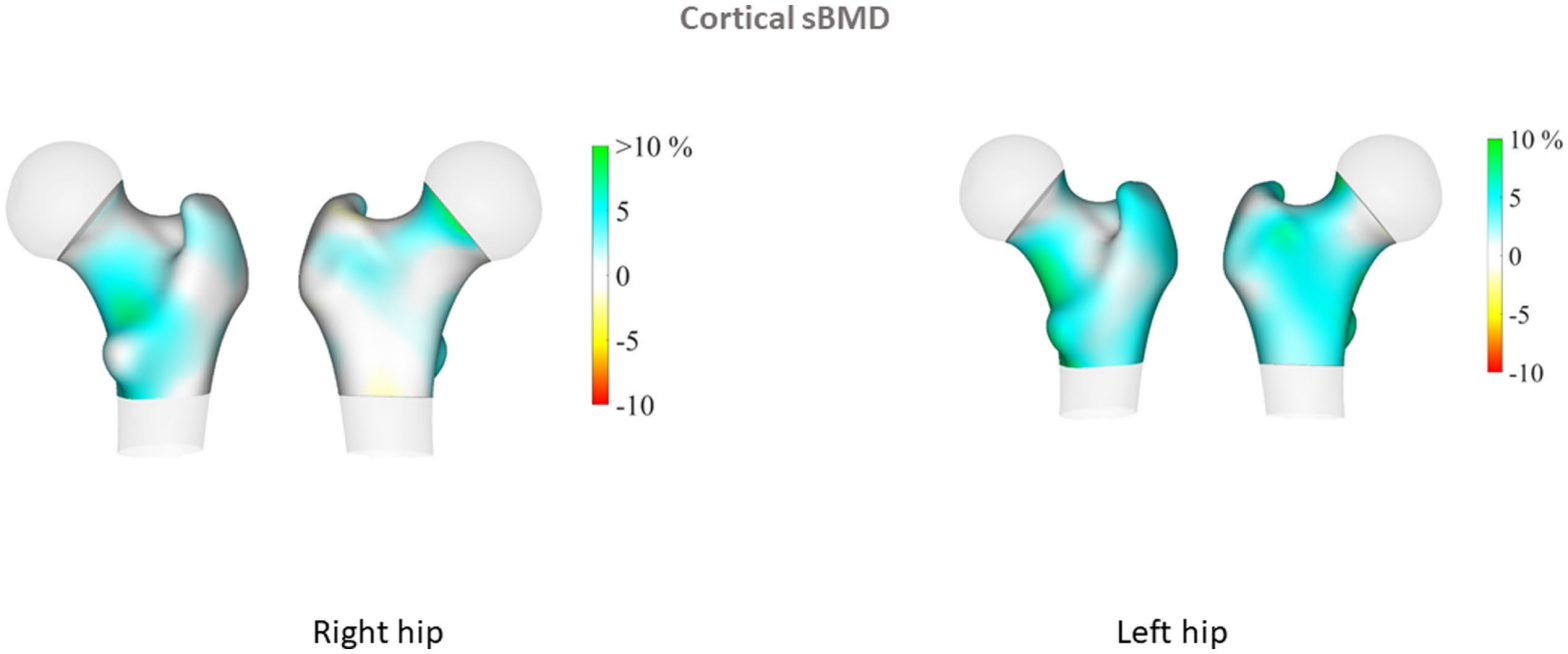
Cortical surface bone mineral density (cortical sBMD). 3D spatial distribution of differences in the cortical bone between the patients with psoriasis (PsO) (left femur *N* = 52, right femur *N* = 52) and their paired controls. Increases in cortical sBMD, vBMD and thickness are presented in blue-green color while decreases are presented in yellow-red color. Results are given for the right and left hips. Each figure shows the anterior and posterior view of the proximal femur. Results illustrate differences in percentage between PsO and controls. DXA-based 3D parameters were higher in patients with PsO, especially for the left femur. Differences were significant for cortical parameters.

**Figure 2:**
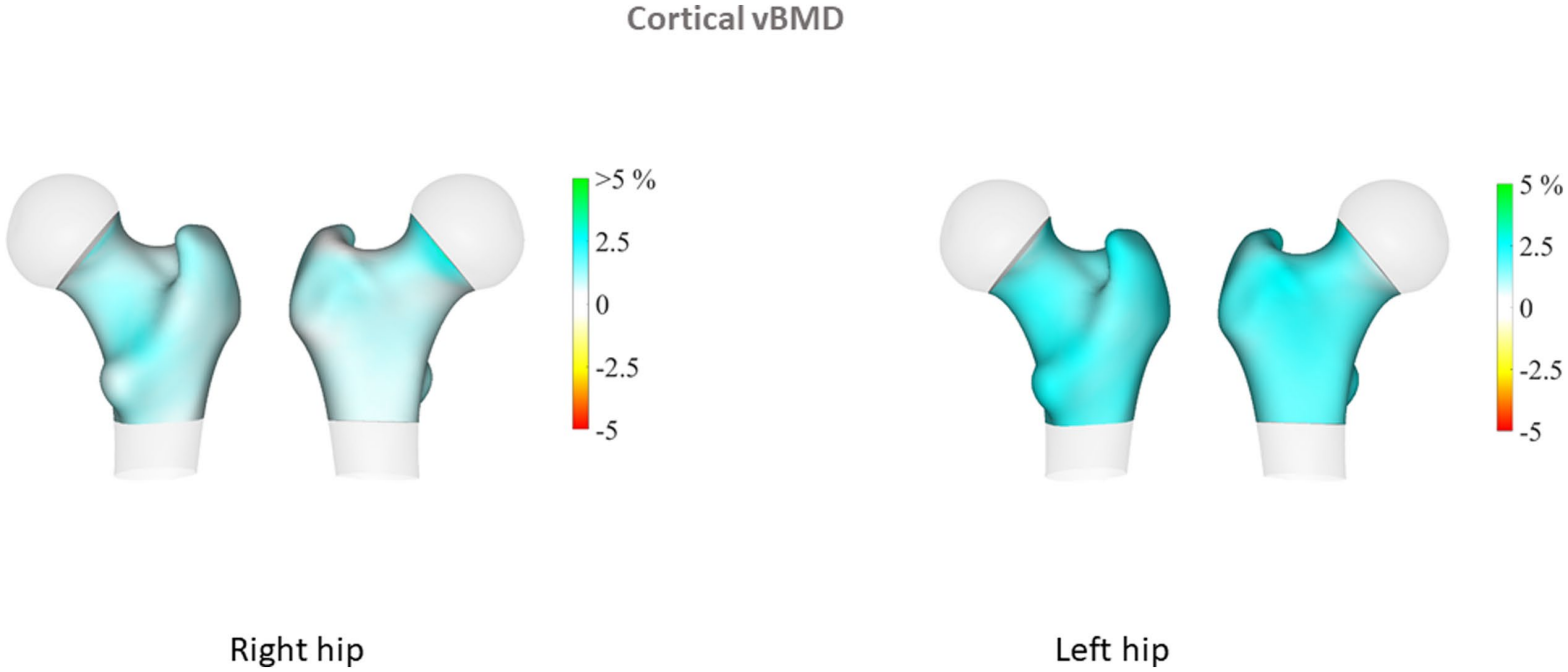
Cortical volumetric bone mineral density (cortical vBMD).

**Figure 3:**
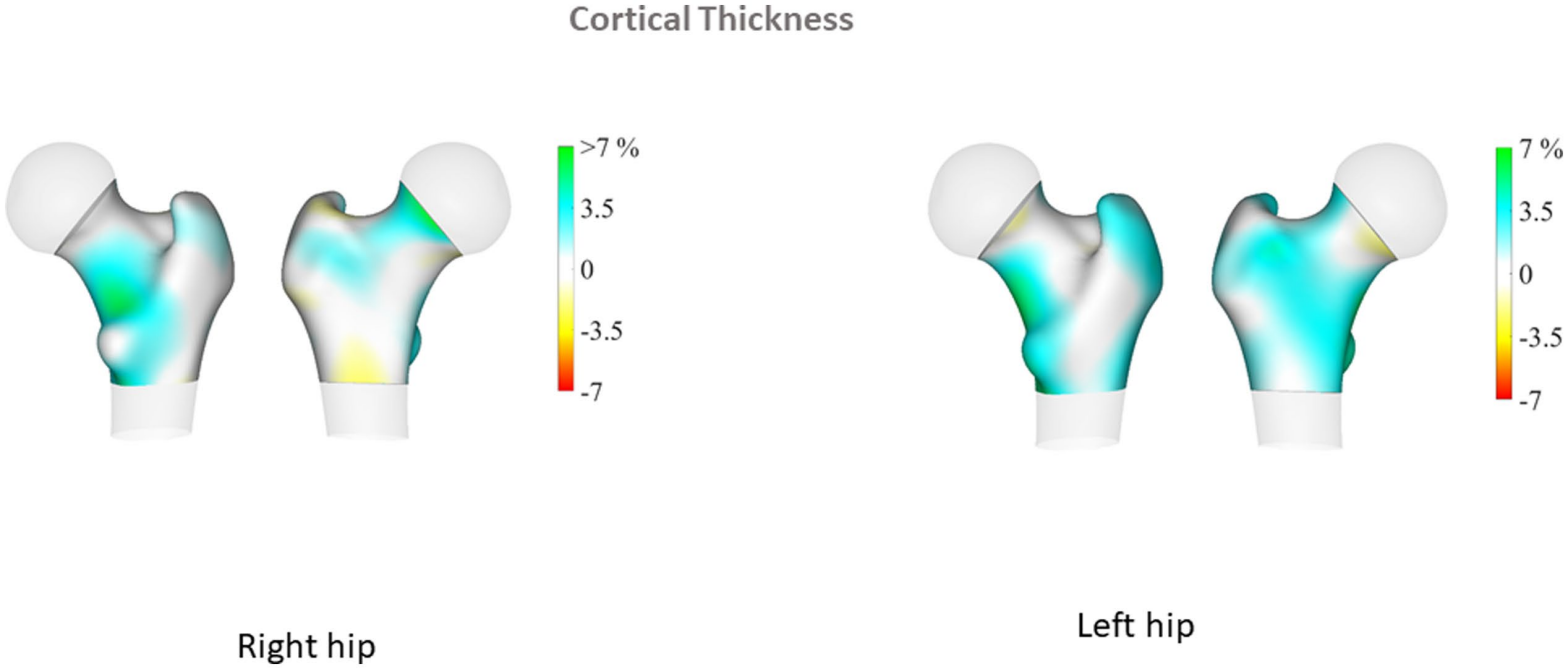
Cortical thickness.

**Figure 4:**
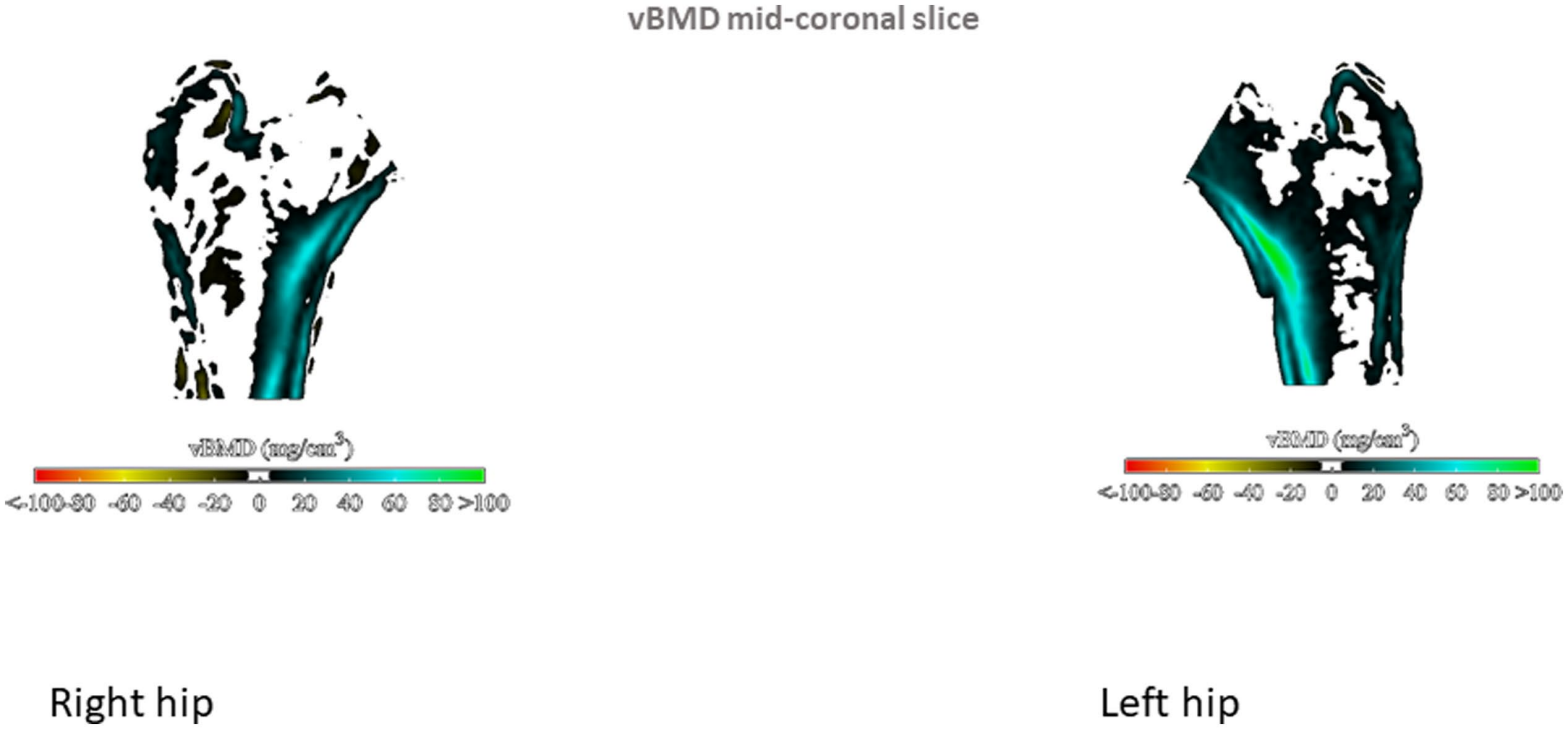
Mid-coronal slices were generated and compared to display changes in volumetric bone mineral density (vBMD).

Proximal femur 3D parameters did not differ between patients with PsA and their controls, either on cortical or trabecular assessment. Conversely, in the PsO group, we observed that TH cortical sBMD of the left proximal femur was significantly elevated in patients compared to their controls (*p* = 0.04). In addition, sub-analysis of the left proximal femur regions showed that cortical sBMD was higher in all regions of the hip (including neck, intertrochanteric and shaft regions) in the PsO group compared to their controls (all *p* < 0.05; Table 2). TH Cth was slightly elevated in PsO patients compared to their controls (*p* = 0.06), and differences were significant for the intertrochanteric and shaft regions (*p* = 0.04 and *p* = 0.03, respectively). On the right side, TH Cth of the PsO group was marginally elevated compared to the controls (*p* = 0.06) and the difference was significant only for the intertrochanteric region (*p* = 0.01). Figures 1–4 show the 3D spatial distribution of differences in the cortical bone between the patients with PsO and their paired controls.

Multiple linear regression analysis was then performed for patients with PsO and their paired controls, adjusting for major confounders (age, gender, BMI and menopausal status). We found that patients with PsO still had higher left FN aBMD, but the difference did not reach statistical significance (*p* = 0.08). Total body aBMD did not differ between PsO patients and their paired controls (*p* = 0.11). TBS remained significantly lower in PsO than in controls (*p* = 0.01), while cortical parameters at the proximal femur obtained from 3D analysis were still higher in PsO patients than in controls: left femur TH cortical sBMD (*p* = 0.05) and TH Cth (p = 0.05). However, when examining the different regions of the left hip for cortical sBMD, the differences for neck, intertrochanteric and shaft regions did not persist. Regarding Cth, intertrochanteric and shaft regions were still higher in PsO patients compared to their controls (*p* = 0.05 and 0.01 respectively). There was no difference in TH Cth of the right femur between patients and controls, even at the intertrochanteric region (*p* = 0.07).

Finally, we examined the association between TBS measurements and markers of disease activity of the patients with PsO. The relationships between bone measurements and disease activity in the PsA group were not examined since both bone mass and bone quality parameters did not differ between the patients and their controls. There were no relationships between TBS and PASI, ESR and CRP while TBS showed a borderline correlation with IL-6 (*r* = − 0.27; *p* = 0.06). DXA measurements of the left femur strongly correlated with 3D-analysis parameters of the left femur (FN aBMD and neck cortical sBMD: *r* = 0.85, *p* < 0.0001; TH aBMD and TH cortical sBMD: *r* = 0.93, *p* < 0.0001; and TH aBMD and TH Cth: *r* = 0.72, *p* < 0.0001).

## Discussion

Our results show that patients with PsA had comparable aBMD at different measurements sites compared to control subjects matched for age, gender and BMI, but also comparable TBS and 3D DXA parameters. Conversely, patients with PsO were characterized by higher aBMD at the femoral neck than their matched controls. In parallel, these patients had higher proximal femur 3D reconstruction parameters compared to their controls, with involvement of the cortical layer but not the trabecular compartment. In addition, only the left femur was involved. Lastly, TBS was unaffected in the PsA group while it was decreased in patients with PsO.

These results are in line with previous evaluations of bone density by DXA in patients with PsA. Indeed, despite initial reports of decreased BMD at the LS, the hip and total body (18), there have been several cross-sectional studies indicating that bone density was not, or only mildly affected in patients with PsA (reviewed in (4)). In addition, a recent population-based study from Norway found that aBMD at the LS and the FN, but not at the TH was higher in patients compared to controls (19). Despite the role played by systemic inflammation and specific inflammatory cytokines on local/periarticular bone loss, as reflected by bone erosions in PsA, it is thought that systemic bone loss and osteoporosis are not major issues in patients with PsA (5, 20, 21). In parallel, we did not find changes in TBS in our PsA patients. There has been no study evaluating the bone of patients with PsA using TBS. Lastly, 3D DXA analysis of the proximal femur did not find altered cortical or trabecular parameters. Bone quality in PsA has previously been evaluated by HR-pQCT: in one study from Germany analyzing the ultradistal radius, trabecular parameters (trabecular BMD, trabecular number and trabecular bone volume fraction) were decreased in patients with PsA compared to a control group (8). In contrast, in a second study from China, only cortical parameters (cortical vBMD, cortical porosity) were found to be lower than in controls (22). The discrepancies between these 2 studies evaluating the distal radius may be explained by ethnic differences in bone microstructure (8). Taken together, it was suggested that bone quality of both the trabecular and cortical compartments may be altered in patients with PsA, thus supporting a bone involvement that may explain a higher fracture risk despite normal aBMD (8). In this study, we only included patients with peripheral arthritis while patients with an axial involvement were not specifically evaluated. Thus, we cannot comment on the bone involvement of this patient subgroup. Osteoporosis has been well documented in patients with axial spondyloarthritis. The pathogenetic mechanisms of bone involvement in inflammatory arthritis has been related to the effects of systemic inflammation but recent data in rheumatoid arthritis underlined the influence of autoantibodies independently of systemic inflammation or disease activity (23). Similar mechanisms may probably influence bone mass in PsA despite the absence of pathogenic antibodies.

In contrast, our patients with PsO were characterized by changes in bone density and quality. Despite some reports of low aBMD in psoriatic patients with a prevalence of osteoporosis around 18% (24), it is acknowledged that osteoporosis and fracture risk are not a major concern in patients with PsO (25). Indeed, in a cross-sectional study in post-menopausal Brazilian women, there were no differences in aBMD at the LS and TH between patients with PsO and their matched controls (26). Data from a population-based study did not argue for an association between osteoporosis, low BMD (at the LS, FN of TH) and PsO (27). This is not in keeping with the results of the large fracture study from a nationwide emergency department sample, which concluded that patients with PsO had a higher odd of fracture [Odd ratio (OR): 2.35; 95% confidence interval (CI): 2.19–2.53]. However, the study recorded pathological fractures based on diagnostic codes, without information on the circumstances of the fracture, and aBMD was not available (6). One study evaluated TBS in patients with PsO alone. However, there was no control group and the study analyzed TBS at baseline and after 6 months of TNF inhibitor treatment, showing no significant change (9). In our series of patients with PsO, TBS was decreased with a mean value of 1.24, which corresponds to the threshold of intermediate risk for osteoporotic fracture (28). In addition, patients with PsO had a longer disease duration compared to patients with PsA and thus disease duration may have influenced TBS results.

The 3D DXA analysis in our patients with PsO showed higher values for cortical parameters than among controls, a result that was significant on the left femur, even after adjustment for major confounders including menopausal status. These results paralleled our DXA results on left FN, and collectively suggest greater hip bone strength. The finding of higher aBMD than control subjects matched for age, gender and BMI has previously been reported for PsO (22, 27) and PsA patients (19). The reasons that may explain higher BMD in patients include demographic differences (age, BMI, menopausal status, smoking) between the patients and their controls, and also the presence of ligamentous ossifications at the spine and/or the hip, an explanation that is plausible only for patients with PsA. In our PsO patients, DXA and 3D DXA parameters were analyzed taking into account major confounders that could introduce bias. However, it remains unexplained why our results were significant only on the left side. We did not register in our patients their dominant side, but it is acknowledged that laterality do not have an influence on bone mass. On the contrary, TBS assessment showed decreased values in PsO compared to controls, suggesting there is alteration of the bone microarchitecture. We previously reported in patients with PsO from the same cohort higher android and visceral fat mass compared to their paired controls (14). The influence of body fat depots on TBS measurements has been previously evaluated, with the demonstration of a negative impact of visceral fat mass on TBS. (29) Thick android fat mass may underestimate TBS but also LS BMD, but it is considered that android fat mass surrounding the LS cannot only explain this negative relationship between TBS and abdominal/visceral fat. Additional studies also reported similar results (30, 31). It has been proposed that adipokines and/or specific proinflammatory cytokines specifically produced by visceral adipose tissue may negatively influenced TBS. Thus, our results of low TBS values in the PsO group characterized by visceral fat mass accumulation are in keeping with previous results in the general population. The discrepancies between TBS and 3D DXA modelling may be explained by the site of assessment, LS for TBS, and the proximal femur for 3D DXA, with different bone compositions (trabecular versus cortical). In addition, TBS is a bone texture surrogate derived from DXA scan of LS, while 3D Shaper is an indicator of bone structure from the proximal femur. HR-pQCT in patients with PsO alone did not find differences regarding vBMD and microstructure of the trabecular and cortical bone between patients and controls (8). However, this study evaluated distal radius and no information about FN or TH was given.

The results of DXA-based 3D modelling showed higher hip cortical bone parameters in the PsO group. These results completed those from DXA examination, providing additional and separate information on trabecular and cortical compartments. Together with the high aBMD at the FN, our results are suggestive of bone quality preservation and consequently, maintained bone strength at the hip. These results are concordant with the population-based study reporting no increased risk of fracture, including hip fracture in patients with PsO [hazard ratio: 1.03 (0.82–1.31)] (26).

The strengths of this study include the fact that patients and controls were adequately matched and well balanced for major confounders. Second, patients did not receive biologic agents, a therapeutic class that may increase bone mass, and a low proportion of them received systemic glucocorticoids. Third, patients had long-standing and active disease, enabling us to evaluate the impact of systemic inflammation on bone mass and microarchitecture. However, some limitations in this study should be noted, namely the cross-sectional design; the small sample size in each group; and the inclusion of middle-aged patients with a low proportion of menopausal women. In addition, we did not specifically evaluate the presence of spinal ossifications (syndesmophytes or osteophytes) at the spine, both in the patient and the control group, but this could influence only LS aBMD measurements. Finally, serum calcium and phosphorus parameters were not evaluated, nor were vitamin D levels.

## Conclusion

patients with PsA had normal aBMD, TBS and DXA-based 3D parameters. Patients with PsO were characterized by higher FN bone density by DXA measurement, and higher cortical parameters by DXA-based 3D analysis, supporting enhanced bone strength and resistance to hip fracture. Conversely, bone texture by TBS assessment was decreased in patients with PsO, and this may contribute to vertebral bone fragility.

## Trial registration

The ADIPSO study (*Évaluation du tissu ADIpeux et des adipokines dans le PSOriasis et le rhumatisme psoriasique et analyze de ses relations avec le risque cardiovasculaire*) is a case–control study conducted in Besançon, France registered on ClinicalTrials.gov under the number NCT02849795.

## Data availability statement

The raw data supporting the conclusions of this article will be made available by the authors, without undue reservation.

## Ethics statement

The studies involving humans were approved by Comité de Protection des Personnes CPP Est-II, reference number: 13/411. The studies were conducted in accordance with the local legislation and institutional requirements. The participants provided their written informed consent to participate in this study.

## Author contributions

ET: Conceptualization, Data curation, Formal analysis, Funding acquisition, Investigation, Supervision, Validation, Writing – original draft, Writing – review & editing. RW: Formal analysis, Investigation, Methodology, Writing – review & editing. FA: Investigation, Writing – review & editing. DW: Investigation, Writing – review & editing. CV: Conceptualization, Funding acquisition, Supervision, Writing – review & editing. MD: Conceptualization, Formal analysis, Methodology, Validation, Writing – review & editing.
